# Different approaches to test orientation of self in space: comparison of a 2D pen-and-paper test and a 3D real-world pointing task

**DOI:** 10.1007/s00415-022-11446-8

**Published:** 2022-11-07

**Authors:** J. Gerb, T. Brandt, M. Dieterich

**Affiliations:** 1 Department of Neurology, University Hospital, Ludwig-Maximilians University, Munich, Germany; 2 Graduate School of Systemic Neuroscience, Ludwig-Maximilians University, Munich, Germany; 3 German Center for Vertigo and Balance Disorders, University Hospital, Ludwig-Maximilians University, Munich, Germany; 4 Hertie Senior Professor for Clinical Neuroscience, Ludwig-Maximilians University, Munich, Germany; 5 Munich Cluster for Systems Neurology (SyNergy), Munich, Germany

**Keywords:** Spatial memory, Spatial orientation, Pointing task, Bedside test, Dementia, Vestibulopathy

## Abstract

Spatial orientation is based on a complex cortical network with input from multiple sensory systems. It is affected by training, sex and age as well as cultural and psychological factors, resulting in different individual skill levels in healthy subjects. Various neurological disorders can lead to different patterns or specific deficits of spatial orientation and navigation. Accordingly, numerous tests have been proposed to assess these abilities. Here, we compare the results of (1) a validated questionnaire-based self-estimate of orientation/navigation ability (Santa Barbara Sense of Direction Scale, SBSODS) and (2) a validated pen-and-paper two-dimensional perspective test (Perspective Taking Spatial Orientation Test, SOT) with (3) a newly developed test of finger-arm pointing performance in a 3D real-world (3D-RWPT) paradigm using a recently established pointing device. A heterogeneous group of 121 participants (mean age 56.5 ± 17.7 years, 52 females), including 16 healthy volunteers and 105 patients with different vestibular, ocular motor and degenerative brain disorders, was included in this study. A high correlation was found between 2D perspective task and 3D pointing along the horizontal (azimuth) but not along the vertical (polar) plane. Self-estimated navigation ability (SBSODS) could not reliably predict actual performance in either 2D- or 3D-tests. Clinical assessment of spatial orientation and memory should therefore include measurements of actual performance, based on a 2D pen-and-paper test or a 3D pointing task, rather than memory-based questionnaires, since solely relying on the patient’s history of self-estimated navigation ability results in misjudgments. The 3D finger-arm pointing test (3D-RWPT) reveals additional information on vertical (polar) spatial performance which goes undetected in conventional 2D pen-and-paper tests. Diseases or age-specific changes of spatial orientation in the vertical plane should not be clinically neglected. The major aim of this pilot study was to compare the practicability and capability of the three tests but not yet to prove their use for differential diagnosis. The next step will be to establish a suitable clinical bedside test for spatial memory and orientation.

## Introduction

Deficits in spatial orientation can occur in both cognitive and vestibular disorders [1]. A navigational impairment is an early symptom of cognitive decline such as mild cognitive impairment or Alzheimer’s dementia. This potentially poses the danger of “wandering-off-behavior” [2]. Early diagnosis requires quantitative testing of patients’ performance in spatial orientation, spatial memory and navigation. Different methods have been proposed to assess these abilities, including psychometric questionnaires [3], paper-based 2D-tests [4] and navigation tasks in virtual [5, 6] or standardized real-world environments [7, 8]. Clinical application of complex tests is rare because of the amount of time and work required. Taking the history of the patients and their relatives will only disclose severe deficits, whereas beginning disturbances require more sensitive objective reliable measures.

Since real-world testing of orientation and navigation capabilities appears impracticable, the question arises whether an easier to perform finger-arm 3D real-world pointing task (3D-RWPT, [9]) is suitable to discern impending spatial orientation deficits. In an earlier study, it was shown that the simple 3D-RWPT delivered reliable and reproducible data on spatial memory in azimuth and polar coordinates [9].

In a first step, this test was now compared to a commonly used and validated questionnaire (Santa Barbara Sense of Direction Scale, SBSODS [4]) and to a 2D paper-and-pencil orientation test (Perspective Taking Spatial Orientation Test, SOT [3]). The participants included 16 healthy volunteers and 105 patients with various degenerative cognitive or vestibular and ocular motor disorders. The major goal at this stage was not to establish criteria for differential diagnosis but to compare the practicability and sensitivity of a 3D real-world test for spatial orientation.

## Methods

### Participants

Sixteen healthy volunteers and 105 patients presenting with different vestibular pathologies, gait instability, central ocular motor disorders, neurodegenerative syndromes or cognitive disorders underwent a standardized 3D-pointing task sitting on a swivel chair and two well-established psychometric tests, a questionnaire (SBSODS) and a 2D paper-and-pencil orientation test (SOT). Out of these 121 participants (mean age 56.5 ± 17.7 years, minimum 24 years, maximum 86 years, 52 female), 50 had either a unilateral (*n* = 23) or a bilateral (*n* = 27) peripheral-vestibular hypofunction, 31 had a cognitive impairment, 25 suffered from polyneuropathy and 9 had ischemic lesions. These groups were not mutually exclusive, i.e., participants with both vestibular and cognitive deficits were included in this analysis, since patient recruitment was part of an ongoing effort to create a large-scale clinical database for future examination of orientation deficits in different neurological disorders. Therefore, the only exclusion criteria were an age below 18 years and the inability to perform the pointing task due to, e.g., joint-neuromuscular disorders, severe visual deficits, pareses or movement disorders. Patients presenting with vertigo or dizziness underwent a complete neuro-otological assessment [10] including caloric irrigation of the semicircular canals, video head impulse test and neuro-orthoptic examination. Healthy participants all had no history of vestibular disorders, vertigo or dizziness and received a clinical neurootological examination including Frenzel’s glasses and head impulse test to rule out a relevant peripheral-vestibular deficit.

The data protection clearance and Institutional Review Board of the Ludwig-Maximilians University Munich, Germany, approved the study (no. 094-10) and all patients gave informed consent. The study was performed in accordance with the ethical standards laid down in the 1964 Declaration of Helsinki and its later amendments.

### Self-report of navigation ability: Santa Barbara Sense of Direction Scale (SBSODS)

The SBSODS consists of 15 questions about the self-perceived sense of direction with replies being registered on a Likert scale with seven intervals (1: fully agree, 7: fully disagree). If subjects left a blank space, an intermediate value of 4 (4: I don’t know) was registered. Overall, a higher score means a higher self-reported sense of direction [4].

The test was initially developed and validated in English and had to be translated for the present study. The recommended cross-cultural adaptation process [11] was followed, namely translation of the original SBSODS—and also SOT—version from the English language into German, synthesis of translations, and back translation into English by a native speaker blinded to the original versions. Translations were then evaluated by the initial developers deciding on equivalence; if necessary, the above-mentioned steps were repeated after retranslation and correction. This process is established in the field of vestibular research [12].

### Two-dimensional perspective taking: Spatial Orientation Test (SOT)

**Fig. 1:**
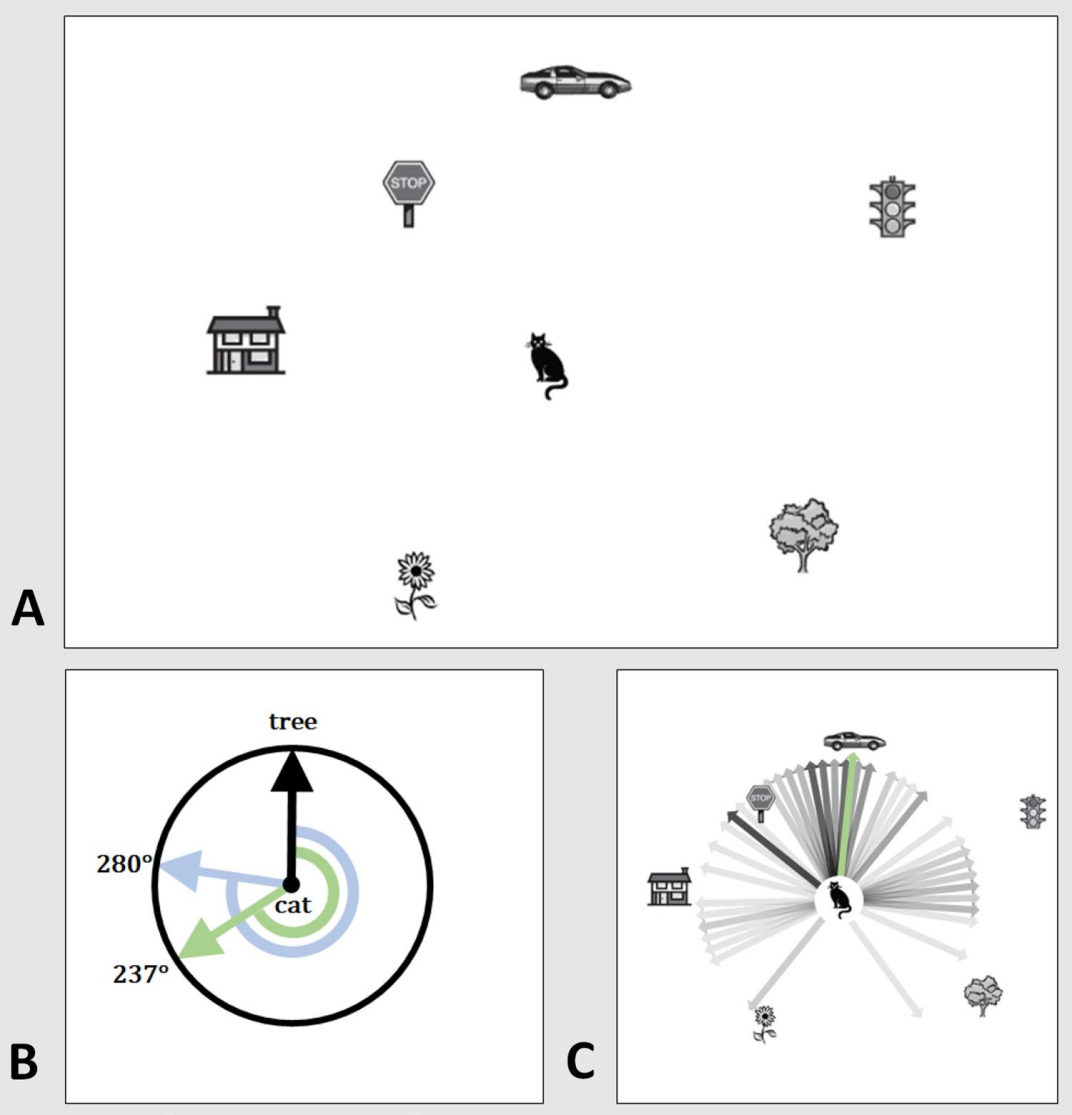
Schematic presentation of the two-dimensional paper-and-pencil test (SOT). **a** Subjects are shown a bird’s-eye-view of a map filled with seven objects and a task, e.g., “Imagine you are standing at the cat and facing the tree. Point to the car.” No annotations or real-world rotation of the test are allowed. **b** The relative direction should be indicated in the circle provided where the object in front of one’s virtual position is already indicated by an upward arrow (correct solution: green arrow and angle: 237°; example participant: light blue arrow and angle: 280°). Participant solutions are measured as mean deviation from correct solution in degrees on a unit circle (in this example: 43°). **c** Virtual rotation of participant solutions back onto the initial map showing large-scale angular deviations up to 170° in few participants (*n* = 104, light to dark gray arrows show participant solutions; darker lines represent multiple participants answering identical directions. Green arrow: correct solution as provided by the original authors). Modified from [3]

The tasks from the original English version of the SOT [3] were annotated with German translations and printed on DIN A4 paper. We chose a paper-based version of the SBSODS and SOT to allow for error-correction and to not add a barrier for less technologically inclined elderly patients. While large-scale reviews could prove the overall good comparability of pen-and-paper and computer-based tests [13], the interaction with digital devices can facilitate negative emotional responses in elderly subjects [14] which is detrimental to subject motivation and might artificially skew testing performance. On each trial of the SOT, subjects are shown a two-dimensional bird’s-eye-view of an unchanged map filled with seven objects (Fig. 1). The test requires the subject to imagine being located at one of the objects, facing a second object and then indicating the relative direction toward a third object from said virtual position. No annotations on the test sheet or physical rotation of the test are allowed. To ensure a sufficient understanding of the task, one sample task was explained by the examiner. Thereafter, subjects had 5 min to solve as many of the 12 tasks as possible. Angular errors of the indicated direction were measured to the closest 5° interval on a 360° full circle; lower errors mean a better performance. The maximum error is 180°, i.e., subjects giving a direction completely opposite of the correct solution. Furthermore, the number of tasks solved in the 5-min time test phase was also determined (nSOT).

### 3D finger-arm pointing test (3D-RWPT)

**Fig. 2:**
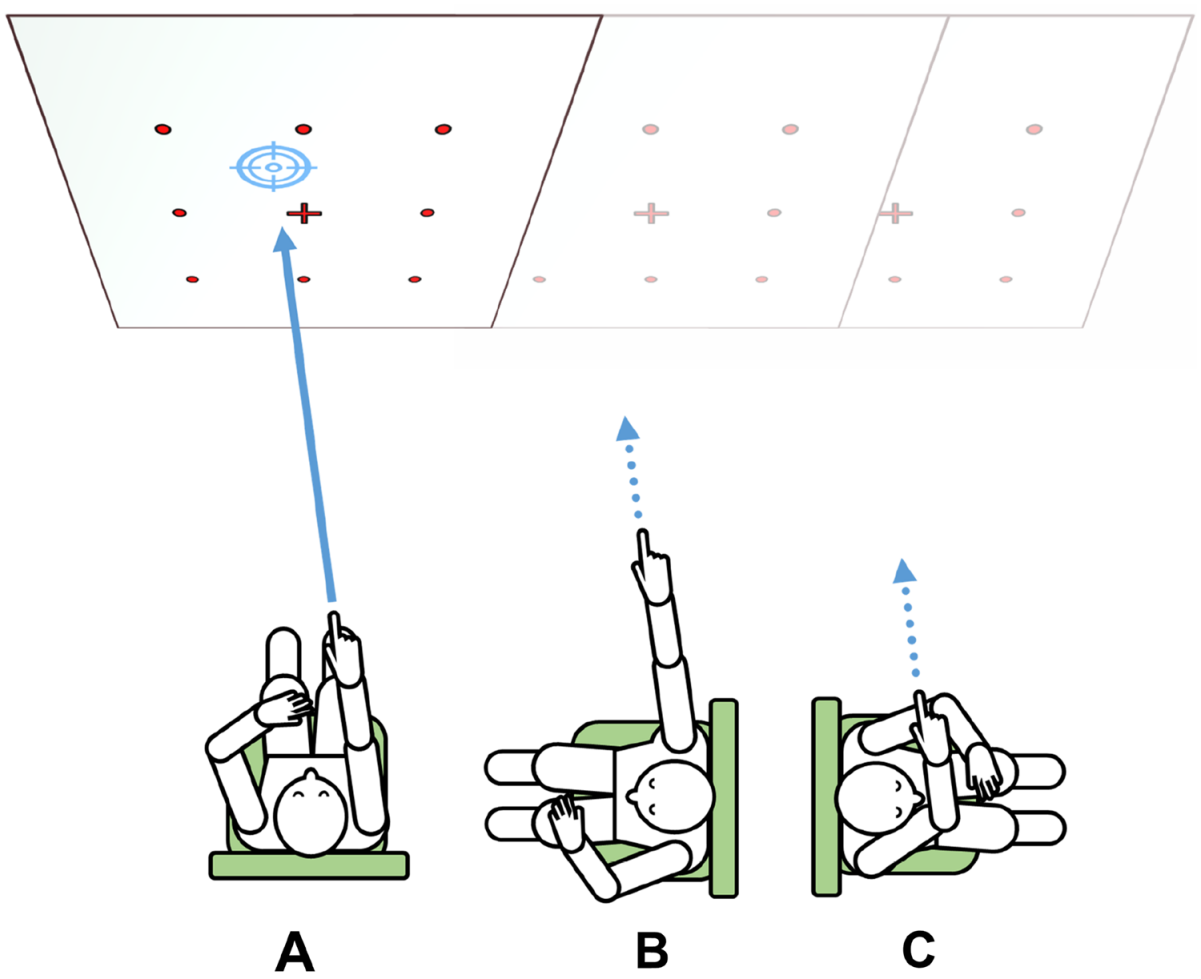
Illustration of the 3D-finger arm pointing paradigm. While the subject with eyes open was seated on a swivel chair in a standardized centered position in front of a white wall with a nine-point matrix marked on it, the pointing device is calibrated to each point in a randomized order. Afterwards, the subject was asked to point to each target in five conditions with the eyes closed and the body in **a** neutral position straight ahead, **b** 90° rotation to the non-dominant side, back in neutral position (**a**), **c** 90° rotation to the dominant side resulting in elbow flexion, and back in neutral position (**a**). The tasks performed in neutral position are used to calculate deviations due to task repetitions, while the performance in position **b**, **c** allow determination of deviations due to spatial transformation of body-to-wall position

The participant was sitting on a swivel chair using a smart phone based pointing device on their dominant hand (testing setup with high spatial discrimination and simple handling; for details see: [9]; Fig. 2). The pointing task consisted of two calibration and five testing paradigms: one calibration for a retinotopic solution where the subject uses an egocentric reference frame and one calibration with a smart phone-fixed laser depicting the pointing trajectory, requiring a world-based allocentric reference frame. During calibration, visual feedback was available. Targets were marked with red 20 mm points on a white wall in a 3 × 3 matrix with a 100 cm distance between points. For each task, a computerized voice from the device gave a command in randomized order, e.g., “top left” in either German or English. The subjects then pointed toward the target with their extended dominant arm and confirmed the final position with a wireless Bluetooth dongle with the other hand. After calibration, the subjects were asked to point to the targets in (newly) randomized order without visual feedback while facing toward them (1), after passive 90° body rotation on the chair to their non-dominant side with visual feedback available during rotation (2), back to the initial body position without visual feedback during rotation (3), after passive 90° body rotation to their dominant side with visual feedback available during rotation (4) and back to the initial target-facing position, without visual feedback during rotation (5). Each test run was separated by a standardized pause of 30 seconds which was signaled in 5 second intervals using a notification sound of the pointing device. Handedness was assessed using the Edinburgh inventory [15].

### Data analysis of 3D-RWPT

Applying the technique described [9], the coordinates from the device accelerometer sensors readout for each target measurement were transformed into vectors in a spherical coordinate system centered on the subject’s position by assuming a stable gravitational acceleration on the *z*-axis. Since the required trigonometric operations for data processing included periodic formulas, deviations larger than *π*/2 (180°) would potentially give wrong results; large-scale deviations were therefore monitored by test personnel. By standardizing and strictly controlling subject position in 3D-space, these vectors can then be compared to both calibrations, resulting in two azimuth and two polar deviations in degrees from retinotopic calibration and from allocentric/world-based calibration, respectively. These deviations were then averaged, resulting in a mean azimuth deviation (mAD) and a mean polar deviation (mPD) from retinotopic and world-based calibration (mAD_retinotopic_, mAD_world-based_, mPD_retinotopic_, mPD_world-based_). Furthermore, the tasks which were performed facing the wall (1, 3, 5) were averaged to create the mean reproduction deviations (Reprod. mAD, reprod. mPD) and the tasks where a transformation from a 90° body rotation position relative to the wall was required (2, 4) were averaged to calculate the mean transformation deviations (Transform. mAD, transform. mPD). Again, deviations from retinotopic or world-based calibrations were calculated (Reprod. mAD_retinotopic_, reprod. mAD_world-based_, reprod. mPD_retinotopic_, reprod. mPD_world-based_, transform. mAD_retinotopic_, transform. mAD_world-based_, transform. mPD_retinotopic_, transform. mPD_world-based_).

### Statistical data analysis

All data were irreversibly anonymized for further analyses and processed using JASP (Jeffreys’s Amazing Statistics Program, Version 0.16.2 [16]). For data description, we used mean values and standard deviation for continuous variables and absolute and relative frequencies for categorical variables. We tested statistical inference using Spearman’s rho (ρ) and used an independent samples Student *t* test for group comparisons.

## Results

Of 121 participants (mean age 56.5 ± 17.7 years, minimum 24 years, maximum 86 years, 52 female), four did not complete the SBSODS but performed the SOT and 3D-RWPT while 11 healthy controls only performed an abbreviated version of SBSODS and SOT. To avoid a lengthy presentation of the numerous data, the results are depicted in Tables 1, 2, 3 and 4. Mean test results and pointing deviations are depicted separately for females and males (Table 1).

**Table 1:** Results of tests in 121 participants (16 healthy volunteers, 105 patients)

	Sex	Valid	Missing	Mean	Std. deviation	Minimum	Maximum
SOT	M	63	4	56.488	32.991	6.833	109.583
	W	47	7	62.972	31.406	6.25	113.917
SBSODS	M	61	6	5.016	0.99	2.867	6.867
	W	45	9	4.481	0.87	2.667	6.867
mAD retinotopic	M	67	0	9.301	4.838	4.39	25.179
	W	54	0	11.507	6.904	4.172	33.189
mAD world-based	M	67	0	10.019	5.986	4.496	40.769
	W	54	0	12.335	8.103	3.275	47.221
mPD retinotopic	M	67	0	7.466	2.629	3.235	14.744
	W	54	0	8.155	2.649	3.407	15.153
mPD world-based	M	67	0	6.681	2.366	3.306	14.698
	W	54	0	6.363	1.472	3.858	9.796
Reprod. mAD retinotopic	M	67	0	7.727	4.237	3.705	23.247
	W	54	0	8.973	5.039	3.014	26.294
Reprod. mAD world-based	M	67	0	8.691	5.913	3.367	34.166
	W	54	0	9.743	6.951	2.707	43.674
Reprod. mPD retinotopic	M	67	0	6.68	2.74	2.654	14.495
	W	54	0	7.29	2.48	2.098	14.318
Reprod. mPD world-based	M	67	0	6.076	2.385	2.234	14.183
	W	54	0	5.745	1.658	2.617	10.049
Transform. mAD retinotopic	M	67	0	11.663	7.374	2.83	31.482
	W	54	0	15.31	12.326	3.574	73.739
Transform. mAD world-based	M	67	0	12.009	7.683	3.828	50.673
	W	54	0	16.222	12.748	3.723	72.791
Transform. mPD retinotopic	M	67	0	8.645	3.017	3.761	17.31
	W	54	0	9.452	3.313	5.113	19.019
Transform. mPD world-based	M	67	0	7.589	2.73	3.935	17.145
	W	54	0	7.29	1.877	3.757	12.914

SOT: mean angular deviation in ° (lower score means higher performance). SBSODS: mean score of self-reported sense of orientation (higher score better). mAD, mPD: mean pointing deviation in ° in azimuth (mAD) and polar (mPD) direction, further subdivided into reproduction tasks (Reprod.) and transformation tasks (Transform.) and deviation compared to retinotopic calibration and world-based calibration, respectively. All results divided by participant sex (M/W)

**Table 2:** Pairwise statistical correlation of participant age and 3D-pointing task in azimuth and polar direction (further subdivided into reproduction tasks (Reprod.) and transformation tasks (Transform.) and compared to retinotopic calibration and world-based calibration), 2D pen-and-paper test angular error (SOT), number of pen-and-paper tasks solved in a 5 min period (nSOT), and self-reported sense of direction (SBSODS), respectively

	Spearman	
	*ρ*	*p*
Age		
mAD retinotopic	0.391***	< 0.001
mAD world-based	0.4***	< 0.001
mPD retinotopic	0.075	0.42
mPD world-based	0.277**	0.002
Reprod. mAD retinotopic	0.289**	0.002
Reprod. mAD world-based	0.302***	< 0.001
Reprod. mPD retinotopic	0.072	0.437
Reprod. mPD world-based	0.319***	< 0.001
Transform. mAD retinotopic	0.381***	< 0.001
Transform. mAD world-based	0.401***	< 0.001
Transform. mPD retinotopic	0.032	0.729
Transform. mPD world-based	0.146	0.115
SOT	0.544***	< 0.001
nSOT	− 0.475***	< 0.001
SBSODS	0.019	0.845

**Table 3:** Statistical correlation (Pearson, Spearman) between self-reported sense of direction (SBSODS), 2D pen-and-paper test (SOT) and the 3D-pointing task paradigms in azimuth direction, further subdivided into reproduction tasks (Reprod.) and transformation tasks (Transform.) and deviation compared to retinotopic calibration and world-based calibration, respectively

		Spearman	
		*ρ*	*p*
SBSODS	SOT	− 0.183	0.061
	mAD retinotopic	− 0.1	0.307
	mAD world-based	− 0.093	0.341
	Reprod. mAD retinotopic	− 0.105	0.286
	Reprod. mAD world-based	− 0.053	0.591
	Transform. mAD retinotopic	− 0.093	0.342
	Transform. mAD world-based	− 0.077	0.435
SOT	SBSODS	− 0.183	0.061
	mAD retinotopic	0.292**	0.002
	mAD world-based	0.375***	< 0.001
	Reprod. mAD retinotopic	0.181	0.059
	Reprod. mAD world-based	0.256**	0.007
	Transform. mAD retinotopic	0.31***	< 0.001
	Transform. mAD world-based	0.411***	< 0.001

**Table 4:** Statistical correlation (Pearson, Spearman) between self-reported sense of direction (SBSODS), 2D pen-and-paper test (SOT) and the 3D-pointing task paradigms in polar direction, further subdivided into reproduction tasks (Reprod.) and transformation tasks (Transform.) and deviation compared to retinotopic calibration and world-based calibration, respectively

		Spearman	
		*ρ*	*p*
SBSODS	mPD retinotopic	− 0.159	0.103
	mPD world-based	− 0.038	0.701
	Reprod. mPD retinotopic	− 0.215*	0.027
	Reprod. mPD world-based	− 0.027	0.78
	Transform. mPD retinotopic	− 0.097	0.322
	Transform. mPD world-based	− 0.024	0.805
SOT	SBSODS	− 0.183	0.061
	mPD retinotopic	0.044	0.651
	mPD world-based	0.103	0.284
	Reprod. mPD retinotopic	0.073	0.447
	Reprod. mPD world-based	0.138	0.149
	Transform. mPD retinotopic	0.022	0.817
	Transform. mPD world-based	0.04	0.678

**Fig. 3:**
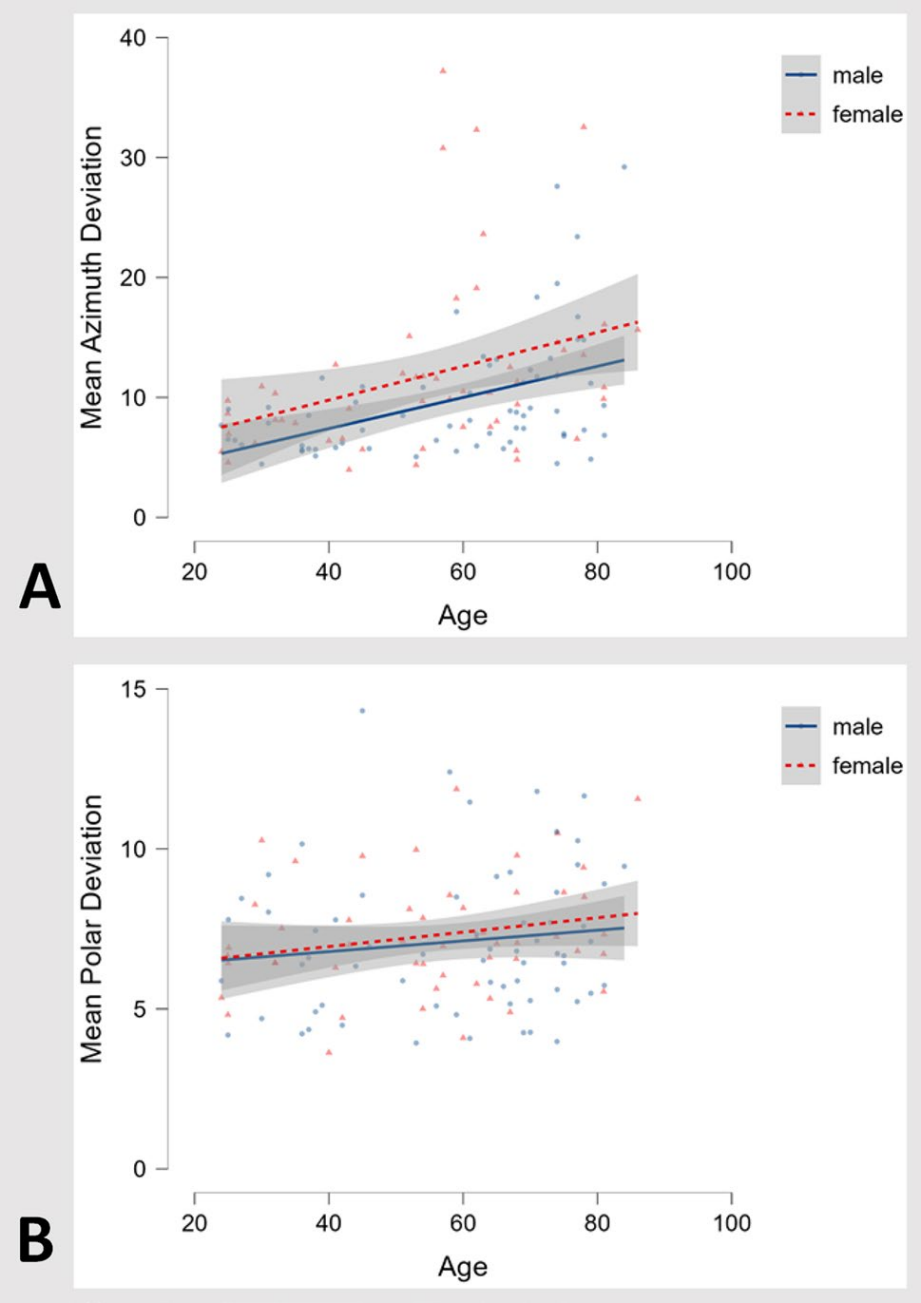
3D pointing task: **a** Plot of mean azimuth deviations (in °, lower scores equaling better performance) against participant age in years for both sexes (male participants: blue line, female participants: red dotted line, *n* = 121). A decrease of performance with age can be seen in both sexes, while male participants across all ages on average performed slightly better than female participants. **b** Plot of mean polar against participant age in years for both sexes, showing no clear effect of either age or sex

Objective 2D and 3D performance as well as self-reported sense of direction showed a slight sex bias in favor of male participants. Higher participant age resulted in worse performance (Table 2). Overall, 3D azimuth pointing performance exhibited a sex and age bias (Fig. 3a), while 3D polar performance was more stable across sex and age (Fig. 3b). An independent samples Student *t* test based on equal variances [*t*(121) = − 1.994, *p* < 0.05; Cohen’s *d* = − 0.365] showed that male participants had a significantly lower mean azimuth deviation (9.660°, SD = 5.122°) than female participants (11.921°, SD = 7.326°), while polar performance did not have a significant sex bias [*t*(121) = − 0.488, *p* = 0.626; Cohen’s *d* = − 0.089, mean male deviation = 7.074°, SD = 2.277°; mean female deviation = 7.259°, SD = 1.792°].

Similarly, 2D pen-and-paper-tests and 3D azimuth pointing correlated considerably (Table 3), whereas 2D and 3D polar performance did not robustly correlate (Table 4).

**Fig. 4:**
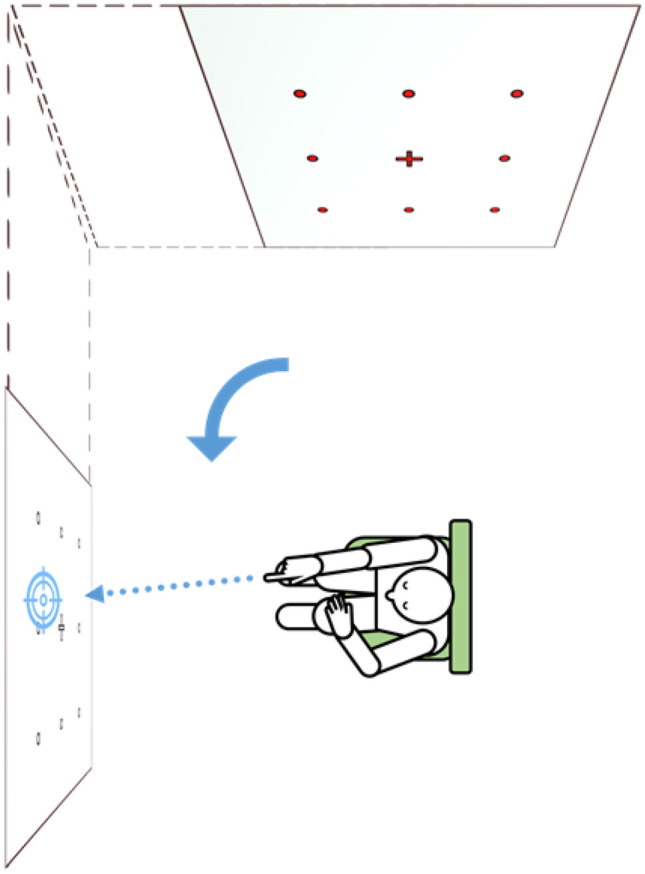
Example of observed “egocentric fallback” behavior where participants point to targets in a remembered egocentric reference frame after body rotation without prior transformation to match the changed relationship between body and environment. This may result in large azimuth deviations, possibly with unaffected indications of target directions in polar coordinates. The rotation was performed actively with auditory and visual cues present

Complete inability of reference system updating was observed in five patients, who assumed the targets to be still in front of them after active rotation to the side (“egocentric fallback”; Fig. 4).

As explained in the Introduction, this pilot study did not aim to differentiate between specific disorders but rather between the methodological capability of different tests. Therefore, we refrained from presenting quantitative data on specific subgroups of disorders.

## Discussion

In this preliminary data analysis, the validated 2D pen-and-paper orientation test and the newly developed 3D real-world pointing task [9] showed significant correlations in a heterogeneous group of participants. Well-known findings of spatial research in a 2D task (e.g., sex bias or age dependence) could be confirmed and further demonstrated for the 3D task. Parametric independent *t* testing of azimuth pointing performance showed a small, but significant difference in favor of male participants (Cohen’s *d*: − 0.365), a finding in line with meta-analyses of sex differences in navigation tasks [17]. While further clinical data is required to investigate disease-specific spatial orientation deficits, the aim of this study was to perform a comparison of 2D and 3D participant performance and explore the value of self-reported navigation ability in both healthy subjects and patients. This was motivated by the clinical observation of discrepancies between an individual’s objective test performance and subjective questionnaire results of individual ability. We could show that both 2D and 3D tests give reliable results of a participant’s spatial performance. In contrast, a self-report of these skills is prone to errors, possibly due to, e.g., overestimation of one’s abilities during the lifelong span of performance [18], increasingly affected by unawareness or denial or palliation of one’s cognitive decline [19]. This fits prior research conducted in healthy subjects, e.g., the low predictive value of the SBSODS overall score regarding a cardinal-pointing task (e.g., directional pointing to north, east, south and west) in its validation study [4] or other real-world-navigation experiments with healthy college students [20]. While the overall SBSODS-score could not predict 2D or 3D test performance in the current study population, only some sub-items of this questionnaire were of predictive relevance. Similar effects have already been reported in a large study focused on spatial abilities in an Italian community [21] which introduced a distinction between positive (e.g., pleasure in exploring new environments) and negative self-assessments (e.g., spatial anxiety). Only positive self-assessments explained pointing accuracy in healthy participants, whereas negative self-assessments did not [20]. A neuropsychological and imaging study on patients suffering from bilateral vestibulopathy, on the other hand, had shown a difference in the negative assessments, namely the Spatial Anxiety Scale [22] while other psychometric tests did not differ between healthy subjects and these patients [23]. The SBSODS overall score includes positive and negative questions, while the Spatial Anxiety Scale can differentiate even further sub-aspects of spatial anxiety [24]. Taken together, the importance of a detailed assessment of subjects’ or patients’ orientation-related history is limited as to the expected skill level, be it with detailed questionnaires or thorough history taking. Thus, for actual performance assessment both 2D pen-and-paper and 3D pointing tests are more suitable measures to uncover deficits of spatial orientation.

While SOT and overall azimuth deviation showed a high correlation, SOT and polar deviation often did not coincide. This might be due to the nature of the SOT which tests two-dimensional directionality similar to azimuth pointing. Furthermore, as a ground-based species, human everyday pathfinding is overall confined to the horizontal plane; it might therefore be a more commonly trained task, possibly especially by active young and healthy subjects. The perception and estimation of horizontal and vertical dimensions of imagined buildings, on the other hand, were shown to be error-prone even in familiar environments [25, 26]. It is unclear whether a direct neuroanatomical substrate of the horizontal or vertical mental representation of one’s three-dimensional environment exists.

Overall, the observed mean polar deviations were lower than the mean azimuth deviations. This might partially be due to the yaw-axis-rotation before the transformation tasks constituting a change of body position only on the horizontal but not on the vertical plane. Further research is required to see if polar pointing performance, possibly utilizing an additional pitch-axis transformation task, allows a distinction of orientational deficits that are currently underrepresented in, e.g., 2D testing batteries.

When comparing the reproduction tasks and the transformation tasks, we could show that the transformation tasks (e.g., after rotation to the side) exhibited a higher correlation with 2D-performance than the reproduction tasks. This could be due to participants applying proprioceptive information (e.g., remembered joint position) in the forward-facing reproduction task, while the transformation tasks necessarily involve reference-system adaptation (from a mental map in either retinotopic or world-based coding toward a shoulder-centered motor plan) and therefore require spatial processing. Further research will be required to see if subjects with visuo-spatial cognitive deficits show distinct deficits in these subtasks.

We noticed a pattern of large-scale deviation in exceptional patients (*n* = 5) who kept applying an egocentric reference frame after rotation to the side, assuming the targets to be still in front of them (Fig. 4), although they had performed the rotation under sensory control by vision (the rotation was performed with open eyes) and hearing (e.g., the command given by the examiner). Further research in a larger group of patients with cognitive deficits will be necessary to improve the understanding of the applied pointing strategies and determine if this “egocentric fallback,” i.e., the faulty direct usage of egocentrically (in our case: retinotopic) coded spatial information without prior transformation to consider changes of the relationship between body and environment, is a disease-specific characteristic.

When analyzing these test results or when rating clinical orientation performance, it is important to keep in mind a subject’s baseline or pre-disease skill level. Multiple factors such as subject age [27] or sex [28] can determine test performance with some developmental studies also showing effects of exercise [29], childhood hobbies [30] or other early-life activities [31]. In studies on targeted locomotion, higher cognitive social inter-personal aspects of interaction with the environment [32, 33] and an individual disposition could be shown to affect results. Wayfinding as both a navigation as well as a decision task is also a social activity and can be influenced by group phenomena, even when other people are not directly present [34]. These factors are well-known in the field of psychological research [20, 35], but are often paid less attention in neuroscientific experiments. Nevertheless, these differences create relevant challenges in the clinical examination of orientation or navigation performance. Simply put, a landscape architect applying three-dimensional navigation and orientation every day comes from a higher level than a desk worker, regardless of the orientational demand. Uncovering disease-specific minor differences outside of overall large-scale performance metrics requires heterogeneous patient groups as well as a simple, yet precise test of spatial navigation. Here, the 3D-real-world pointing task might be able to disclose new disease-specific characteristics in future studies.
